# Enhanced Transport Capabilities via Nanotechnologies: Impacting Bioefficacy, Controlled Release Strategies, and Novel Chaperones

**DOI:** 10.1155/2011/902403

**Published:** 2011-04-26

**Authors:** Thomai Panagiotou, Robert J. Fisher

**Affiliations:** ^1^Microfluidics International Corporation, P.O. Box 9101, Newton, MA 02464, USA; ^2^Chemical Engineering Department, Massachusetts Institute of Technology, Building 66, Room 305, 77 Massachusetts Avenue, Cambridge, MA 02139-4307, USA

## Abstract

Emerging nanotechnologies have, and will continue to have, a major impact on the pharmaceutical industry. Their influence on a drug's life cycle, inception to delivery, is rapidly expanding. As the industry moves more aggressively toward continuous manufacturing modes, utilizing Process Analytical Technology (PAT) and Process Intensification (PI) concepts, the critical role of transport phenomena becomes elucidated. The ability to transfer energy, mass, and momentum with directed purposeful outcomes is a worthwhile endeavor in establishing higher production rates more economically. Furthermore, the ability to obtain desired drug properties, such as size, habit, and morphology, through novel manufacturing strategies permits unique formulation control for optimum delivery methodologies. Bottom-up processing to obtain nano-sized crystals is an excellent example. Formulation and delivery are intimately coupled in improving bio-efficacy at reduced loading and/or better controlled release capabilities, minimizing side affects and providing improved therapeutic interventions. Innovative nanotechnology applications, such as simultaneous targeting, imaging and delivery to tumors, are now possible through use of novel chaperones. Other examples include nanoparticles attachment to T-cells, release from novel hydrogel implants, and functionalized encapsulants. Difficult tasks such as drug delivery to the brain via the blood brain barrier and/or the cerebrospinal fluid are now easier to accomplish.

## 1. Introduction

A large number of hydrophobic compounds with potentially high pharmacological value fail to pass initial screening tests because of the perception that they will be too difficult to deliver effectively due to anticipated formulation limitations. Fortunately, nanosuspensions of such drugs may be used to increase bioavailability and offer a variety of delivery options. Historically most formulation strategies aim for particle size reduction [1–4]. Typically these limit the dimensions obtainable since the strategies use high shear processing of preformed entities. To achieve nanoscale dimensions by these size reduction technologies (“top down” processing), an excessive amount of energy and time needs to be expended [5, 6]. Unfortunately, they often not only proved ineffective but lead to possible product degradation. Because nanosuspensions and novel targeting chaperones, for example T-cells, can deliver much larger amounts of drug in a smaller volume than the solvent diluted drug systems [1–4, 7–9], they have a potential advantage as a formulation strategy.

Emerging nanotechnologies are having a major impact throughout the pharmaceutical industry. The focus here is on how these techniques influence delivery strategies and efficacy through enhancement of the transport phenomena involved in all phases of a drug's life cycle. For example, the ability to obtain desired drug properties, such as size, habit, and morphology, through novel manufacturing strategies permits unique formulation control for optimum delivery methodologies. The ability to transfer energy, mass, and momentum with directed purposeful outcomes is imperative in establishing higher production rates of these carefully engineered nanoparticles at elevated technoeconomic stature. 

The role of transport phenomena becomes critically apparent as the industry moves more aggressively toward continuous manufacturing modes, utilizing Process Analytical Technology (PAT) and Process Intensification (PI) concepts. Although these advances rely upon more effective sensor-reporter systems, based on nanoprobe technology, they are not the focus here and therefore will only be briefly touched upon in the following discussions. The emphasis is on the clinical aspects that drive all the other phases needed to get to this stage. That is, once available, these nanoscale entities can be utilized quite effectively in both traditional and novel delivery techniques, relying heavily on *in vivo* transport capabilities. The topics to be addressed in the following sections all capitalize on how carefully these drugs were designed, developed, and engineered for desired properties and capabilities. Specificity of uptake, clearance control, and transport to the brain via the blood brain barrier, cerebrospinal fluid, or in smart implants are a few examples.

Currently, there are a number of nanotechnology drugs in the market [10]. This first generation of such drugs relies mainly on the small size of the particles to increase the surface area and therefore bioavailability of poorly soluble drugs, and to a lesser extent in the structure of the particle for delayed release, and so forth. Examples of nanotechnology drugs in the US market include Rapamune^®^/Pfizer, Emend^®^/Merck, INVEGA^®^ SUSTENNA^®^/Janssen, all based on Elan's NanoCrystal^®^ technology. Abraxane^®^/Abraxis Bioscience and Triglide^*™*^/Sciele Pharma are also in the US market. In emerging technologies, the particles have improved functionalities that include diagnosis, targeting, and drug delivery functions and enhance transport and uptake characteristics. The focus of this paper will be in these emerging technologies rather than the current status of the market drugs.

The credibility of the techniques (topics) being presented here is established through either prior extensive testing, preliminary results from proof-of-concept tests, or derived from analogous successes for what are believed to be realistic projected applications. Presented here therefore will be discussions relative to (a) crystal size and morphology control, via bottom-up processing, for direct use with traditional delivery methods, (b) simultaneous targeting/delivery techniques incorporating novel chaperones obtained from functionalized surfactant encapsulants and T-cells, and (c) controlled release using nanotechnology innovations involving single and multiple drug interventions and tissue therapies (e.g., angiogenesis, wound healing, and artificial organs for autoimmune diseases). In these cases, attempts are made to identify the underlying fundamental physicochemical principles/mechanisms associated such that projected extensions are feasible, and scaleup where necessary can be accomplished reliably.

## 2. Techniques/Applications

In the recent article by G. Liversidge [10], as mentioned previously, a number of specific pharmaceutical companies and associated drugs are identified that combine control-release and nanotechnologies. This combination is identified as a key market driver for this industry. Based upon documented recent advances and successful applications, various potential opportunities are outlined. Powerful extensions to many of the concepts and methods mentioned there are being developed and some are currently being implemented throughout the industry. For example, the concept of minitablets has a profound impact on many release formulations, (i) delayed-, (ii) extended-, and (iii) pulsitile-release systems. 

An objective of ours via this paper is to identify the importance and effectiveness of nanotechnological innovations on the enhancement of transport processes that improve therapeutic protocols. Of the techniques being discussed, the bottom-up method for nanocrystal formation will be used as an example because it provides the basis for our ability to carefully engineer the nanoparticles for the drug delivery protocols. These entities are an essential component for the clinical implementation of all the transport enhanced techniques in use and/or proposed. Whenever available, the results from the various levels of experimental programs executed are presented and discussed, conclusions drawn, and recommendations for future efforts set forth.

Presented in Table 1 below is an outline of the current and emerging methods and nanotechnology applications in drug delivery platforms. These topics will be discussed or referenced in the sections that follow. 

### 2.1. Formation of Engineered Crystalline Nanoparticles

A continuous bottom-up approach to the solvent/antisolvent crystallization process allows precise control of product properties. Achievement of specified quality goals associated with overall performance criteria has been demonstrated [11–14]. These include crystal habit, morphology, and size distribution. The technique involves generating a large number of nucleation sites and limiting subsequent growth. With this method crystal size control is via molecular approaches that utilize various mechanistic pathways governed by transport phenomena, thermodynamics principles, and/or intrinsic kinetics. 

The design and operation of commercial scale crystallizers are optimized based on minimizing the formation of agglomerates, impurities included within crystals, liquid entrapped within crystal aggregates, and mother liquor retained by the crystal cake [15–17]. The various crystallization mechanisms that contribute to the observed phenomenological events and how they affect these objectives will be addressed throughout this section.

The generation of nanoscale homogeneous regions dispersed throughout the active crystallization volume is essential for the success of this bottom-up process. Estimating the size of these regions is reasonably straightforward using proven turbulence calculation algorithms [18–20]. The significance is that the length scale over which no further mixing takes place is established and thus molecular diffusion now dictates timing for the steps involved in the homogeneous nucleation and growth processes within these regions. Since hydrodynamics has a significant impact upon mass, energy, and momentum transport rates and reaction proficiency it is imperative that the role it plays not be underestimated. It is also essential to identify the energy dissipation mechanisms present and thereby quantify the intensity of mixing (i.e., macro-, meso-, or micro-), contact efficacy, and associated level of turbulence with its resultant eddy cascade. The length scale of the Kolmogorov (i.e., smallest) eddies, when formed at high energy dissipation levels, can easily be at the nanoscale. The important point is that the magnitude of this energy dissipation rate per unit volume establishes both the time and length scales over which events occur. These can be key control variables manipulated by mixing intensity once the thermodynamic state of the working fluid is established through other processing variables. Observed rates are highly dependent on the concentration differences beyond the solubility limit and hydrodynamic scales. Hence, the local degree of supersaturation can be used as the primary metric to account for both the kinetics and thermodynamic behavior of the system [11, 12, 21, 22].

Crystal characteristics, such as crystal size distribution (CSD), surface area and topography, morphology, dissolution rate, and strength (affected by any impurities and flaws present), depend heavily upon their formation processing conditions. An inclusion of mother liquor for example affects not only product quality for its desired applications but also storage stability, particularly with respect to CSD and morphology. This is of considerable importance to the pharmaceutical industry since polymorphic systems exhibit different physicochemical properties due to the existence of these different crystal structures. Polymorphism influences the dissolution characteristics, which along with CSD affects product formulation strategies and bioavailability [1, 2, 11–14, 23–26]. 

To understand how to form crystalline nanoparticles of hydrophobic active pharmaceutical ingredients (APIs) via this bottom-up process requires knowledge of the fundamental thermodynamic and rate processes involved in the generation of solid particles from a liquid phase. This involves solubility limits of the target species (with associated degree of supersaturation), nucleation and growth rates, and turbulence intensity to obtain the requisite mixing levels. It is the energy dissipation levels developed by turbulence that determine the appropriate length and time scales required to control the phenomenological events occurring. Although these topics are discussed in some detail for specific applications elsewhere [11–22], a brief summary of each is included here for clarity of purpose. 

The various aspects and important parameters that affect the “bottom-up” crystallization process to be discussed are the following.

- *Thermodynamics; *describes phase characteristics, solubility limits and phase stability, establishing the driving force for crystallization.
- *Nucleation and crystal growth; *related to crystallization rates, particle sizes, and crystal structures.
- *Complications; *describes some of the issues that need to be addressed in designing a process and getting the desired product quality.
- *Flow Patterns, Mixing, and Transport Phenomena; *describes the role of mixing in crystallization processes, relevant to processes that involve mixing of multiple streams, heating or cooling.
- *Creating Nanoscale Entities; *describes strategies of achieving mixing in the nanometer scale and techniques used.
- *Energy Dissipation; *gives an overview of the mechanisms that absorb energy during the process.

#### 2.1.1. Thermodynamics

**Figure 1:**
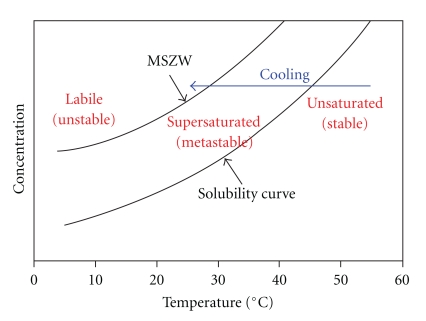
Solubility Curve and Metastable Zone plotted against temperature and concentration.

Generating solids from a liquid phase is initiated by changes in the thermodynamic state of the solution, thereby reducing the solubility of the target species. Initiation may be through temperature adjustment(s), concentration changes, or by altering solution activity coefficients as in the solvent/antisolvent method. Phase stability is an important factor in determining both when and how fast events progress. The Temperature-Composition phase behavior, see Figure 1, can be used to illustrate some important concepts. A solubility curve represents thermodynamic equilibrium between the phases. For most liquid systems with a composition and temperature above its solubility curve a stable unsaturated liquid exists. Beyond this solubility limit the liquid may not be in thermodynamic equilibrium with respect to the formation of the solid phase, that is, it exists as a supersaturated (SS) liquid. System behavior can be determined by this degree of SS since there is a region, referred to as the metastable zone, where the system may not always be considered thermodynamically unstable. Heterogeneous nucleation sites are thus necessary to initiate the formation of the solid phase. However, beyond the boundary of this metastable zone, these seed nucleation sites are no longer required. In this region a SS liquid is neither stable nor in equilibrium, and is subject to spontaneous nucleation and rapid growth of the solids. 

Unfortunately, due to the large increase in entropy, some undesired events may occur. The crystal matrix may have flaws, such as dislocations, impurity molecules, or liquid inclusions. When a system exhibits various polymorphs, this spontaneity could be problematic or beneficial, depending on the morphology sought and its stability. Since our objective is to create a large number of nucleation sites and thereby restrict the ultimate size of the individual particles, and possibly control morphology, this unstable zone is the desired initial operational region. To control the nucleation and growth rates, the strategy used must establish the desired supersaturation state, level of energy input, and energy dissipation mechanisms. The need for the latter two will be discussed in subsequent sections.

#### 2.1.2. Nucleation and Growth

The degree of supersaturation influences the rate of the individual steps involved in forming the solid as well as which crystal polymorph is formed. In general, the process proceeds as follows: (1) feed streams are mixed in a process unit selected to meet required specifications for the energy dissipation rate per unit volume. The time to achieve homogeneity is dependent on diffusivity of the target species and the distance they must travel within the smallest eddies obtained (see the discussion on mixing for the role of turbulence and the Kolmogorov scale); (2) mixing to obtain the desired local degree of supersaturation, leading to a nucleation rate, which increases proportionally with SS. The features of the product formed depends significantly on this rate; (3) growth of the nuclei is by diffusion of solute molecules from the bulk solution to the surface and then along the surface to be integrated into the matrix. This continues until a limiting particle size is reached, determined by the magnitude of the shear force present; (4) further growth is by mechanisms whereby particles collide and adhere to each other. Particle number thus decreases with time as the particle size increases. 

The ability to create and control a supersaturation driving force is paramount to having a robust process. It can be generated by various methods including indirect cooling, evaporation, adiabatic evaporative cooling, antisolvent addition and salting out, chemical reactions, and pH adjustment. Note that temperature changes may be detrimental for some systems, for example when dealing with protein-based drugs. Alternative methods most frequently used to reduce solubility are pH adjustment to the isoelectric point, increasing ionic strength, addition of nonionic polymers, and addition of a miscible nonsolvent. 

#### 2.1.3. Complications

Many factors can restrict productivity and purity. Of particular interest for the bottom-up approach are agglomeration, liquid inclusions, and inefficient mother liquor removal.

**Agglomeration**

The particle size can clearly be affected by agglomeration and fracture mechanisms. When growing crystals collide they may stick together and form new particles, that is, agglomerates form when the collisions are inelastic. The strength of the physical bonds thus formed determines their stability upon further collisions. For the bottom-up processing to be effective in limiting crystal size the probability of agglomeration needs to be low. Unfortunately, a large number of small particles are produced when operating in the unstable supersaturation region and collision frequency is high. To offset this concentration effect, it is necessary to limit the time for interaction and/or relieve SS quickly. Also, a surfactant may be effective in limiting the probability that the particles will stick to one another.

**Liquid Inclusion in Individual Crystals and Agglomerates**

This is particularly undesired when liquid impurities are present. High growth rates can contribute to increased amounts of liquid entrapped within a crystal. Also, liquid can get trapped between colliding particles during agglomeration and higher supersaturation levels increase the probability of that occurrence. Thus high supersaturation can have both beneficial and problematic outcomes. One can mitigate any associated problems by limiting the interaction time and/or relieve the supersaturation condition rapidly.

#### 2.1.4. Flow Patterns, Mixing, and Transport Phenomena

Mixing at the nanometer scale occurs as reactants, which may include several liquid and solid phases, are subjected to high shear stresses and turbulence. The energy dissipation rate determines whether the macro-, meso-, or micromixing level is attained. The overall mixing process occurs within a flow field continuum which covers the wide range of length and time scales indicative of each of these mixing levels, each with distinct characteristics. For example, consider two miscible fluids. The large scale distribution by flow patterns that causes gross dispersion is considered macromixing. Next, the breakdown of large eddies into smaller ones via the “eddy cascade” is termed mesomixing. Fluid engulfment in small eddies with subsequent laminar stretching of them, where molecular diffusion is now the final mechanism to obtain uniform composition, is referred to as micromixing [18–20]. The length scale for this diffusional process is determined by the size of the smallest eddies formed and is referred to as the Kolmogorov length scale. Along with time and kinetic energy scales, each determined by these local flow conditions alone, (i.e., related to kinematic viscosity and the energy dissipation rate per unit mass), the so called Kolmogorov scales are established. Estimating the magnitude of these Kolmogorov parameters can be accomplished with reasonable confidence using proven theoretical turbulence calculations. The significance is that the length scale over which no further mixing takes place is established and molecular diffusion now dictates timing for the necessary steps involved in the homogeneous nucleation and growth processes. 

These mixing subprocesses generally occur in series, but often to some extent, in parallel. Turbulent energy dissipation rates, for example in modified impinging jet technologies [11, 12, 27–29], are estimated to be on the order 10^7^ W/kg and higher when using these micromixing models. At these levels, rapid micromixing and mesomixing (on time scales of 4 and 20 *μ*s, resp.) are achieved, and the length scale of the smallest eddies are at the nanoscale. Note that residence times in many of the microreactors systems used for PI applications [30], particularly those utilizing impinging jets, are of the order 1 ms and lower. 

Incorporating these fundamental principles and using appropriately designed equipment it is possible to precisely control each step in the crystallization process. Mixing at the nanometer scale provides a uniform supersaturation ratio. The onset of the nucleation process can be manipulated by controlling the timing and location of the mixing of the solvent and antisolvent streams that are used to generate the supersaturated state. This in combination with an evenly dispersed homogeneous supersaturation ratio results in uniform crystal growth and stabilization rates.

#### 2.1.5. Creating Nanoscale Entities

The generation of nanoscale homogeneous regions dispersed throughout the system is a major requirement for the success of this bottom-up process. When accomplished, it is reasonable to consider these regions as nanoreactors. This concept is ideal for our purposes since both length and time scales are quite small for the processes involved in creating these monodispersed nanoparticles. Consequently, it is immaterial whether or not these regions are stabilized, as for example, by use of surface active agents. 

It is important to reiterate that the length scale over which no further mixing takes place is established and molecular diffusion now dictates timing for the necessary steps involved in the homogeneous nucleation and growth processes. In the absence of seed crystals or other nucleation sites, a critical number of molecules must collide and remain aggregated forming stable clusters, (i.e., nuclei). Subsequent growth requires diffusion to and along the surface, followed by a specific integration process that incorporates these molecules into the crystal matrix of a particular polymorph. The observed crystallization rate is, therefore, highly dependent on length scales and the local degree of supersaturation. The polymorph that is obtained is dependent on thermodynamic considerations, such as component activity coefficients (solvent/antisolvent/solute species interactions, composition/concentrations, and temperature) and entropy generated due to the spontaneous nature of the process, that is, rates influenced by supersaturation ratios. 

To generate the high energy dissipation rates used to produce nanoparticles, many processing techniques utilize high shear fields. Jet impingement, on a solid surface or with another jet, has been shown to be a highly efficient method [11, 12, 27, 28]. Systems that incorporate high velocity linear fluid jets that collide can rapidly reduce the scale of segregation between the streams. High-energy dissipation is observed because the kinetic energy of each stream is converted into a turbulent-like motion as the result of the collision and redirection of the flow within a very small volume. More thorough discussions on the phenomenological events, equipment design criteria, and characterization studies are given elsewhere [11–15, 18–26].

#### 2.1.6. Energy Dissipation

Surface tension and various molecular forces between the species present are key variables associated with the crystal size distribution. Thus, surface active agents can play a significant role whether as a contributor to growth mechanisms or as a size stabilizer. For example, they are involved in self-assembly mechanisms, and can act as barrier components that restrict transport, as possible chaperones that target specific sites during drug delivery, as sequestering agents to facilitate contact efficacy, as promoters of interfacial phenomena, and as inhibitors to agglomeration. 

The fraction of the input energy available for formation of surfaces is instrumental in establishing system efficacy. Performing an energy audit to determine overall requirements is an essential task for this systems analysis approach. This entails determining the amount of input energy transformed into kinetic energy of the jets, identifying all forms of dissipation (whether desired or not), and ascertaining the amount stored as internal energy. Although the system energy requirements are not readily identified *a priori*, the total energy input and the amount dissipated and stored are measurable. Estimates of the various losses occurring can be made, and the energy utilization for the desired processes can also be estimated. This permits energy considerations to be used in predicting performance from the estimated length and time scales obtained. System validation is accomplished when these length and time scales can be corroborated with observed kinetics phenomena [12, 27, 28].

#### 2.1.7. Examples of Successful Applications

Confined impinging jet systems have been used in our laboratory to consistently produce submicron API suspensions via a continuous process that involves crystallization via the solvent/antisolvent technique to generate supersaturation conditions. Microfliudics Reaction Technology (MRT) was selected for this bottom-up processing since it is based on novel multiple stream inlet capabilities coupled with the impinging jet concept [11–14, 26]. It is designed to produce jet velocities and energy dissipation orders of magnitude higher than those of conventional impinging jet reactors. The technology provides precise control of the feed rates, and the subsequent location and intensity of mixing of the reactants. It may provide significant technical and economical advantages due to its process intensification character that minimizes energy requirements, and the proven scalability of the reactor.

In our first proof of concept studies performed, nanosuspensions of several APIs were produced varying the key parameters of the technology [14]. Five different model APIs were used for testing and were selected to belong to different chemical families that exhibit different pharmacological activities. There were two antibiotics (*azithromycin *and *API-2*), an antihistamine (*loratadine*), an anticonvulsant (*oxycarbazepine*) and a non-steroidal anti-inflammatory (NSAIS, *API-1*). The particle size depended on the supersaturation ratio and energy dissipation expressed as process pressure. The nanosuspensions were stable with narrow particle size distributions and median particle sizes in the range of 50–760 nm. This “bottom up” process was compared to a “top down” process in which drug nanosuspensions were created as a result of particle size reduction. It was found that the “bottom up” process was substantially more efficient and resulted in smaller particles. 

This first study did not attempt to identify crystalline structure and therefore no polymorph selectivity capabilities were evaluated. To accomplish this, two additional, more in depth studies were conducted on single APIs: Carbamazepine (CBZ), an anticonvulsant, and Norfloxacin (NFN), an antibacterial agent. The details of the experimental protocols and results are reported in separate papers, CBZ [12] and NFN [11]. A few brief comments are given here to help validate the benefits of bottom up processing with respect to the stated objectives of creating carefully engineered particles with “tunable” characteristics. 

The NFN nanosuspensions had narrow particle size distributions and median particle sizes in the range of 170–350 nm depending on the supersaturation ratio and energy dissipation expressed as process pressure. However, the particle size was found to be insensitive to the presence of the surfactant used. The crystalline structure of NFN was not affected by the processing conditions for this particular solvent/antisolvent system, but it was different than the initial crystalline structure of the drug. This implies the product is tunable.

The particle habit was needle-shaped. Two miscible fluids were used as the solvent (DMSO) and antisolvent (water). The effect of process pressure (determining the energy input), the NFN concentration, the supersaturation ratio, and the presence of surfactant on the particle size and the crystallized material was investigated. Higher pressures resulted in smaller particle sizes, as did lowering NFN concentration and supersaturation ratios. The surfactant that was used (Solutol) did not affect the particle size. The crystalline structure was not affected by the shear rate of the process. It was identical to those formed in a beaker under low shear conditions. However, the crystallite size of the material decreased threefold from no shear to high shear conditions.

CBZ was selected as a model system since it is known to exhibit polymorph multiplicity. Several solvents and antisolvents were used to determine their effect on the crystalline structure and particle size. CBZ is also known to form hydrates, therefore both aqueous and nonaqueous solvent/antisolvent systems were used for comparison. They were Dichloromethane (DCM)/Hexane, Poly(ethylene-glycol) (PEG) 300/Water, and Dimethyl sulfoxide (DMSO)/Water. 

The results obtained with respect to processing conditions are consistent with those of the NFN study. Particle sizes obtained with all bottom up experiments were consistently in the range of 250–320 nm. Unfortunately, the results obtained with respect to polymorph selectivity were not as definitive. What was observed is that the solvent/antisolvent system does matter, but it is unclear if the degrees of supersaturation or processing intensity had significant roles in that study. Three different morphologies were detected via XRD patterns and a hypothesis is given to explain the detailed observations presented there. Although not conclusive and thus more thorough studies must be performed, the explanations are consistent with those results. 

Although the emphasis in the previous paragraphs was in crystallization, other processes can be used to manufacture nanosized materials with tailored properties. Encapsulation of functional ingredients in polymers is another method, which will be discussed in more detail in the sections that follow.  Table 2 summarizes the processes used in the bottom up production of nanoparticles and the properties controlled via such methodologies.

### 2.2. Simultaneous Targeting/Delivery Techniques

Creative advances in nanotechnologies, coupled with systems biology, has led to novel chaperone systems for simultaneous targeting/delivery, and in certain instances, enhanced controlled release strategies. The systems selected for illustration here are (1) polymer nanosuspensions, (2) functionalized designer surfactant encapsulants, and (3) attachment to T-cell surfaces.

#### 2.2.1. Polymer Nanosuspensions

The creation and use of chaperone systems in targeting, drug delivery, and diagnostic imaging has greatly broadened the applications, and thus needs, for polymer nanosuspensions. The enhanced surface to volume ratios provides unique capabilities for functionalization of the surface for these high degrees of specificity requirements. 

The intended use of these nanosuspensions dictates control of both the mean particle size and distribution. These parameters determine performance and toxicity through the selectivity and rate of receptor-ligand interactions and/or the ability and rate of cellular uptake. The implementation of systems that can control nanoscale phenomena is required and has been reported previously [13]. The techniques reported there can create nanosuspensions of many different polymer types with varying particle sizes by controlling the formulation and process variables. These nanosuspensions may also contain encapsulated species via either co-precipitation or other less efficient cargo loading techniques that rely upon diffusional uptake strategies. 

Encapsulation of active pharmaceuticals and contrast agents within these biocompatible polymers is readily accomplished using bottom-up techniques for co-precipitation processes that are reproducible and scalable. Nanosuspensions in the range of 50–500 nm with different polymers with high encapsulation efficiencies have been created successfully. For example, suspensions of poly(epsilon-caprolactone) (PCL) (a polymer that has been extensively used for parenteral drug delivery) were created using MRT (as discussed above in previous sections). By mixing a 20 mg/mL (PCL/acetone) solvent stream with water at a ratio 1 : 10 (solvent/antisolvent) a nanosuspension with a mean particle size of 220 nm was prepared. Their size and spherical habit was confirmed using SEM instrumentation.

#### 2.2.2. Functionalized Designer Surfactant Encapsulants

There has always been an active interest in targeted drug delivery to tumors to specifically kill cancer cells. Ongoing research in this area has provided significant advances due to the ability to carefully engineer both the vesicle, for its specificity and imaging characteristics, and its cargo API. 

A collaborative team has developed a highly adaptable amphiphilic alternating copolymer system that self-assembles into micelles for therapeutic delivery applications in cancer [8, 9]. The synthetic scheme includes the enzymatic polymerization of multifunctional linker molecules (dimethyl 5-hydroxyisopthalate) with poly(ethylene glycol). This chemoenzymatic synthesis is much faster and more convenient than an entirely chemical synthesis. Subsequent synthetic steps have been developed to attach ligands (for targeting), perfluorocarbons (19F MR imaging), fluorescent dyes (NIRF imaging), and radioiodine (nuclear imaging and radioimmunotherapy) to the backbone polymer. 

Attachment of hydrocarbon or perfluorocarbon side chains provides amphiphilicity to produce the multimodal self-assembling micelles. Additionally, encapsulation procedures for chemotherapeutic agents, that is, doxorubicin and paclitaxel, have been established. These unique alternating copolymer micelle nanoparticles were designed as delivery vehicles targeted to human cancer cells expressing the underglycosylated mucin-1 antigen, which is found on almost all epithelial cell adenocarcinomas, by use of the peptide EPPT, or the folate receptor (FR) by using folate.

Development of the synthetic schemes has been coupled with *in vitro *toxicity tests using various cell viability assays to minimize the toxic effect of these copolymer structures. The nontoxic polymers were brought forward into drug delivery and uptake experiments. Cell death due to doxorubicin increased with encapsulation in these alternating copolymers. Additional slight improvements were observed when targeting ligands were attached to the encapsulating polymer. Similar results were obtained with paclitaxel as the cargo. 

Cellular uptake determined by 125I or 3H radioactive analysis and fluorescence confocal microscopy was also investigated in other *in vitro *studies. Microscopy images of the labeled polymer alone demonstrated that the polymer was most likely confined to vesicles within the cytoplasm and not found in the nucleus, whereas encapsulated doxorubicin was shown to be largely confined to the nucleus. Theoretical models of polyvalent binding were employed to guide the design of the targeting polymers. Unfortunately, the polymers used in this study appeared largely nonspecific for the targeted cells when studied *in vitro*. However, the versatility of these polymer constructs suggests that continuing to optimize for a targeting delivery system for drugs and imaging agents using this polymer platform could be extremely beneficial.

#### 2.2.3. Attachment to T-cell Surfaces

Before discussing the specifics of the use of T-cells in drug delivery protocols, a few general comments about the underlying principles are appropriate. The basis of this approach is attributed to the new, burgeoning field of biohybrid materials which will have a significant impact on the efficacy of drug delivery. This is in addition to their obvious use in bioimaging, cellular functionalization, immune system and tissue engineering, and cell-based therapeutics where cell-environment interactions are critical.

 Of particular interest here are synthetic materials systems such as magnetic micromanipulators, nanoparticulate cellular patches, and functional cell backpacks [31, 32]. These offer exciting possibilities for symbiosis between synthetic building blocks and native biological behavior. The key is the ability to systematically modify the surface of living cells. This was clearly demonstrated by the collaborative efforts of the Cohen and Rubner research groups [31] with functional polyelectrolyte multilayer (PEM) patches attached to a fraction of the surface area of living, individual lymphocytes. These cells remained viable, and with patches containing magnetic nanoparticles the cells could be spatially manipulated using a magnetic field. Since the patches did not completely occlude the cellular surface from the surrounding environment a functional payload could be attached without interfering with the cells ability to perform its native functions. This initial work has led to what is now referred to as cellular “backpacks”, nanoscale thickness, micrometer-sized, photolithographically patterned heterostructured multilayer systems capable of noncytotoxically attaching to the membrane of a living cell. It is interesting to note that these “backpacks” can play an integral part in tissue engineering applications, such as in cell aggregate self-assembly [32] which will be discussed briefly in a later section. 

To illustrate the use of this concept in a drug delivery scenario, an extension of this technique was exploited as follows. In a recently published study, a method of attaching carefully engineered nanoparticles to the surface of T-cells was identified [7]. Although their application was for a cell therapy approach, the T-cells were used as chaperones for the stimulant drugs. They designed drug carrying nanoscale vesicles with lipid characteristics for coupling with the sulfur containing molecules on T-cell surfaces. In their study the researchers injected these cargo carrying cells, each with approximately 100 vesicles loaded with interleukins IL-15 and IL-21, into mice with lung and bone marrow tumors. Once reaching the tumors these packets gradually degraded releasing the drugs over a period of one week. Their concept was for the drug molecules being released to reattach to these chaperone T-cells, stimulating them to replicate and thus provide the requisite tissue therapy. The techniques proved successful in that within 16 days, all tumors in the mice treated in this fashion disappeared and these mice survived for the entire 100-day experiment. Mice that received no treatment died within 25 days and those that received either T-cells alone or T-cells with injections of interleukins died within 75 days. 

A few details of their procedure are presented here to stress the relatively straight forward nature of these protocols and instill confidence that the proposed clinical applications can be realized with a high degree of certainty. Their method exploits the fact that T-cells, like many cell lines, have high levels of reduced thiol groups on their surface, and thus stable coupling of the synthetic drug carrying nanospecies to them is possible. Specifically, liposomes and liposome-like synthetic entities 100–300 nm in diameter, with a drug loaded core and phospholipid exterior layer, were linked to the cells via the thiol reactive maleimide head-groups. A simple two-step process achieved the desired conjugation. The donor cells were first incubated with nanoparticles to accomplish the thiol-maleimide coupling. This is followed by *in situ* conjugation to thiol-terminated poly ethylene glygol (i.e., PEGylation) to quench the residual reactive groups to ensure that only about 20% of the surface thiol groups were involved with the initial coupling, that is, linked with approximately 150 nanoparticles. Stable, nontoxic linkages to live cells were thus accomplished with particles ranging from simple liposomes to complex multilamellar lipid nanoparticles or lipid coated polymers. This benign behavior was anticipated since only 3% of the surface of a typical 7 *μ*m diameter T-cell would be blocked by 200 nm diameter particles occupying 150 sites. 

These results suggest therapeutic cells are promising vectors (chaperones) for actively targeted imaging and drug delivery. Furthermore, the attached entities can be engineered for controlled release of individual or multiple drug sequencing capabilities. What can be envisioned is the use of different vesicles with specific transport or degradation properties or a vesicle composed of, for example, multiple polymeric materials, as will be discussed in the following section devoted to release strategies.

### 2.3. Controlled Release Using Nanotechnology Innovations

For a large number of health care/wellness interventions the controlled release of therapeutic agents is a necessary strategy. Carefully designed API formulations can accommodate a broad spectrum of requirements. The release concepts employed range from (i) simplistic steady release rates {via dissolution, etc.}, (ii) intermittent timed release, (iii) programmed simultaneous and or sequential release of multiple species {antigenic drugs and adjuvants}, to (iv) smart systems responding to stimuli: including single and multiple drug interventions and tissue therapies (e.g., angiogenesis, wound healing, and artificial organs for autoimmune diseases). The applications discussed in the following sections demonstrate the breadth of nanotechnologies that impact these release strategies. These all capitalize on how carefully these drugs were designed, developed, and engineered for desired properties and capabilities. Specificity of uptake, clearance control, and ability to perform extremely difficult tasks, such as drug delivery to the brain via transport across the blood brain barrier, the cerebrospinal fluid, or in smart implants, are highly desired capabilities. Coupling advanced materials development and processing techniques with nanoscience and technology creates innovative opportunities not only for traditional drug delivery capabilities, but helps establish the impact platform technologies necessary for tissue engineering/therapy methodologies.

#### 2.3.1. Passive Delivery Mechanisms

 These traditional schemes are governed by classical thermodynamic and transport phenomena principles. They are highly dependent upon the physicochemical properties and geometric features of a drug's formulation. In addition to solubility limits, size distribution, habit and morphology (when applicable), compaction or encapsulation technique, and diffusivity/mass transfer coefficients are significant contributors to accomplishing a successful therapeutic event. For example, nanosized APIs are more readily distributed uniformly with an excipient and/or adjuvant. They also exhibit greater dissolution rates than larger sized entities having the same total mass of drug retained within the product matrix. These methods utilize the dissolution capabilities of the entrapping matrices. Variable release rates can easily be obtained using a composite structure; each layer having different transport properties. The design of release protocols for multiple APIs, sequenced for optimum efficacy and synergism, is thus straightforward. Furthermore, nontherapeutic layers can be included to (i) provide a delay mechanism, (ii) possibly be a barrier for protection until arrival to the desired local or organ system, and/or (iii) be a sacrificial layer containing an adjuvant or other functional component that would, for example, pre-condition the microenvironment [33]. These techniques have been well documented and need not be reiterated here. Obvious extensions to these methods are incorporated into implant systems with hindered diffusion capabilities, in addition to facilitated delivery due to targeting features. Demonstrated implementations of a few of these, along with some conceptualizations are presented below.

#### 2.3.2. Functionalizing for Specificity and Facilitated Delivery

Novel nanomaterials are designed to possess unique features using molecular engineering concepts. Innovative drug delivery protocols have evolved capitalizing on these and recognizing the analogous processes present during successful applications in related areas. Understanding the binding properties and characterization of transport mechanisms within modified hydrogels and biomembranes [34] provides the bases for designing implants with entrapped vesicles and the controlled release of their cargo APIs. Included here is the concept of pulsitile—release systems [10]; that is, the drug is released as bolus pulses in well defined time intervals (see later section referring to future opportunities for additional comment).

Therapies that require the sequencing of multiple drugs can therefore be accomplished by logical extensions. As examples; (i) amphoteric core-shell microgels, that is, contraphilic two compartment colloidal particles [35, 36] could be used as smart systems; either as implants or chaperones, (ii) the concept of chaperones within a larger vector could also prove feasible; to minimize clearance of the smaller entities, or their catabolism, prior to their uptake at difficult to reach sites such as to the brain and subsequent transport across the blood brain barrier, and (iii) stimulate angiogenesis through release of multiple cytokines (growth factors) from nanovesicles entrapped in functionalized hydrogel beads used as immunoprotective barriers for tissue therapy applications [37–41]. Additional details with respect to the research studies involved in formulating these extensions and conceptualizations can be found in the following sections.

**Transport and Drug Delivery through the Blood-Brain Barrier and Cerebrospinal Fluid**

There are multiple barriers in the central nervous system that inhibit API therapies. The blood-brain barrier (BBB) and blood-CSF (cerebrospinal fluid) barriers are vascular in nature, whereas the other, the brain-CSF barrier, exists between brain tissue and the CSF. The wall of the cerebral microvessels in the brain parenchyma constitutes the BBB. Due to its unique structure it maintains very low permeability to water and solutes. The multicell layer present in the middle of the brain parenchyma is known as the blood-CSF barrier. Present there are ventricular cavities (ventricles) filled with CSF secreted by the epithelial cells of the choroid plexus, a highly vascular tissue with leaky, fenestrated capillaries covered with ependymal epithelium, which has relatively tight junctions. The third barrier, the interface between the CSF and brain tissue, is unlike the other two tight blood barriers since it is relatively leaky. Since it does not prove to be a significant resistance to mass transport it is a probable route for drug delivery once the transport issues with the other barriers are resolved. Given that the area of the BBB is about 1000 times that of the blood-CSF barrier, it is more important to circumvent its impermeability, and therefore that is the focus for continued discussion [42]. Furthermore, since it is not considered as limiting as compared to the BBB, further discussions related to CSF transport are not given here but can be found elsewhere [43].

The transport of substances from capillary blood into the brain tissue is dependent upon molecular size, lipid solubility, binding to specific transporters, and electrical charge [44]. Compared to the peripheral microvessel wall, the additional structure of the BBB and tighter endothelial junctions greatly restricts transport of hydrophilic molecules through the gaps between the cells, that is, the paracellular pathway of the BBB [45]. In contrast, small hydrophobic molecules such as O_2_ and CO_2_ diffuse freely across plasma membranes following their concentration gradients, that is, the transcellular lipophilic diffusion pathway. The BBB permeability to most molecules can be estimated on the basis of their octanol/water partition coefficients. For example, diphenhydramine (Benadryl), which has a high partition coefficient, can cross the BBB with relative ease, whereas water-soluble loratadine (Claritin) is blocked. However, the octanol/water partition coefficients do not completely reflect solute transport. Some solutes with low partition coefficients easily cross the BBB by active or facilitated transport mechanisms, which rely on ion channels, specific transporters, energy-dependent pumps, and a limited amount of receptor-mediated transcytosis. Small drug molecules analogous to glucose, amino acids, and small intermediate metabolites, for example, reach brain tissue via facilitated transport mediated by specific transport proteins, whereas larger molecules, such as insulin and other protein type therapeutic agents, are carried across the BBB via receptor-mediated or adsorptive transcytosis. Furthermore, some small molecules with high octanol/water partition coefficients are seemingly blocked. Thorough data analysis suggests that they are actively pumped back into the blood by efflux systems. For instance, members of the adenosine triphosphate-binding cassette family of exporters are potent energy-dependent transporters. They contribute greatly to the efflux of xenobiotics and due to this protective role impede the delivery of therapeutic agents. Consequently, to develop effective and efficient methods for drug delivery to the brain through the BBB, it is imperative to control its permeability. This requires understanding the mechanism by which these structural components, as well as transporters, receptors, efflux pumps and other components at the endothelium and astrocyte foot processes determine it.

Various methods such as intracerebral implantation, microdialysis, convection-enhanced distribution (CED), osmotic shock, and chemical modification of the BBB have been developed for delivering drugs into the brain. However, the applications of these methods are limited and they can only partially keep with the demands of modern therapies. For instance, the efficiency of intracerebral implantation, microdialysis and CED methods are low since their major transport mechanisms are diffusion and convection of interstitial fluid. For effective treatment of CNS diseases, an adequate amount of therapeutic agents must reach the specific regions of the brain. As discussed earlier, functionalized target chaperones have this ability. They can directly deliver therapeutic agents via these transporters by closely mimicking their substrates, or conjugating the drugs to ligands of the specific surface receptors expressed for transcytosis (receptor-mediated transcytosis, RMT-Trojan horse approach). Furthermore, these functionalized target chaperones are used in delivering cationized proteins, peptides, and as nanoparticle carriers for adsorptive mediated transcytosis (AMT).

Although the exact mechanisms of RMT are not fully understood, the development of drug delivery protocols using receptor targeting has been successful [46–50]. This physiological approach is often referred to as the molecular Trojan horse approach since the therapeutic compounds are delivered to specific sites for transcytosis by various forms of vector carriers. This approach also improves the drug loading capacity. The technique is very promising, but unfortunately there remain a number of hurdles to overcome [48–50]. In particular, even if the total amount of drug transported to the brain is large, most of it may not be efficacious since it might remain associated with brain microvessel endothelial cells and not reach the brain parenchyma. If drug translocation is accomplished by conjugation with an antibody, there exists the challenge of dissociation due to the high affinity of antibodies. Furthermore, specificity for uptake in the brain may be compromised since the BBB receptors utilized there could also have a widespread distribution on peripheral organs; in effect, resulting in a seemingly nonspecific uptake. Not only will this limit efficacy, but could induce additional toxicity.

**Improvements in Encapsulation Technologies for Tissue Therapies**

The success of an implant protocol utilizing entrapped tissue for a therapeutic intervention is highly dependent upon controllability of transport characteristics and the microenvironment [33]. Improving the oxygen supply to encapsulated insulin producing cells has been selected for illustration. The basic concepts are to improve the permeability of the encapsulating hydrogel and maintain a high oxygen partial pressure in the surrounding microenvironment. A number of approaches have been suggested, with some tested and validated [51]. Those that utilize nanotechnology, with their inherent improvement qualities, are the focus in this section. The results of two independent studies that address the individual concepts mentioned above will be discussed briefly. When coupled they should provide a synergistic response. Permeability enhancement was accomplished by entrapping a perfluorocarbon nanoemulsion within the hydrogel capsule [51]. Oxygen supply to the capsule surfaces was enhanced through greater vascularization in the microenvironment by stimulation of angiogenesis by cytokines released from the implant [37–41]. Use of cargo-loaded functionalized nanovesicles that control individual cytokine release rates is an obvious extension to that work. One important goal of these angiogenesis studies was to quantitatively evaluate the rates at which different individual growth factors (GFs) are released from their hyaluronic acid hydrogel implants. The ability of added amounts of heparin to specifically regulate basic fibroblast growth factor (bFGF) or vascular endothelial growth factor (VEGF), release from their gels without loss of ability to stimulate a neovascularization response was investigated both *in vitro *and *in vivo*. For both of these growth factors, the rate of release declined monotonically with increasing heparin (Hp) content. As little as 0.03% w/w Hp significantly moderated the time course of release, while inclusion of 0.3% Hp resulted in sustained release over several weeks [40].

The results of that study suggest the possibility of delivery of growth factors in specified sequences at regulated rates, simply by controlling the composition of the gels. Inclusion of as little as 0.3% Hp in the gels led to significant differences in the rates of release of individual GFs. By taking advantage of those differences, it may be possible to design implants that are capable of both storing and providing sustained, localized *in vivo* release of the growth factors, without loss of their biologic effectiveness.

Co-delivery of a combination of more rapidly released GFs together with more slowly released factors may then permit engineered control of desired physiologic processes such as angiogenesis through use of this selective release sequence concept.

The Johnson et al. study [51] is an example that illustrates the usefulness of permeability enhancement, through nanotechnology techniques, for delivery of tissue based therapeutic agents. Their efforts were to enhance the performance of a bioartificial pancreas to treat diabetes that uses microencapsulation as an immune barrier for transplanted islets of Langerhans. Unfortunately, the barrier also imposes oxygen diffusional limitations that can result in loss of viability and function. It is critical that the necessary amount of oxygen be delivered to encapsulated tissue after transplantation in order to maintain normal levels of insulin secretion. Without a solution that allows for effective oxygen delivery, transplantation of encapsulated tissue may never be successful.

Their investigation included methods to reduce oxygen transport limitations by enhancing encapsulant oxygen permeability, for example, by combination of a highly concentrated perfluorocarbon (PFC) nanoemulsion with alginate (PFC alginate). A theoretical reaction—diffusion model was used to predict the three-dimensional distribution of oxygen partial pressure in a spherical microcapsule and a planar slab containing islet tissue, from which the loss of cell viability and the reduction in insulin secretion rate are estimated. Numerical simulations were carried out for normal alginate and PFC alginate to examine the effect of surface oxygen partial pressure, capsule diameter, slab thickness, and the size and density of dispersed islet tissue. Results show that hypoxic conditions can be reduced, thereby enhancing islet viability and substantially maintaining insulin secretion rate when the PFC nanoemulsion is incorporated in the encapsulation material for both geometries.

The approach was also evaluated experimentally, and the ability to enhance encapsulated tissue survival and function was successfully demonstrated, both *in vitro *and *in vivo. * Intact islets encapsulated in normal alginate and in PFC alginates having the composition described in the numerical predictions were used as model systems. Recovery of viable tissue after culture under various O_2_  partial pressure conditions was expressed as the oxygen consumption rate (OCR)/unit volume of capsule divided by the same parameter measured immediately after encapsulation and before culture. When cultured at very low pO_2_, fractional OCR recovery was substantially greater with PFC alginate than with normal alginate. Furthermore, examination of histological sections revealed necrosis in some islets in normal alginate capsules cultured at 3.5 and 142 mm Hg, whereas no necrosis was observed in islets within PFC alginate capsules. The findings and insights gained from both the theoretical and experimental studies will increase the probability of a successful cell therapy for the treatment of diseases such as diabetes.

The concept of “backpacks” discussed earlier with respect to drug chaperones can also be applied to encapsulation techniques and tissue therapies. The commonality rests with the use of nanofabrication approaches to create these entities, for example, the photolithographic method reported previously [31, 32]. The product of this manufacturing step can be either the cell-backpack complexes or freely suspended backpacks. Since these backpacks can carry a myriad of compounds with differing functionalities, their applications seem boundless. Of particular interest here with respect to tissue engineering is the ability of these freely suspended backpacks to promote cell aggregate self-assembly. The size of these aggregates, as influenced by backpack diameter and ratio of cells to backpacks in the culture medium, has been shown to be reproducible [32]. Furthermore, the binding strength is quite strong; which was demonstrated by forcing the complexes through small pores and noting that the backpacks were not removed from the surface of the cells. The importance lies in the ability to use injection techniques (as in a needle tip of a syringe assemble), or for the movement from blood to tissue (extravasation) via narrow gaps. Based on these successes, one can envision applications that would create organoids of various types, such as lymphoid and beta cell clusters (analogous to islet of Langerhans). In these cases, the cargo could consist of drugs, adjuvants, and/or growth factors (for angiogenesis stimulation, reproduction, etc.). There also appears the potential for wound healing protocols.

To support our conjectures, some specific results should be elucidated. In their paper [32], the Cohen group presents fundamental studies on forming cellular aggregates using injectable cellular backpacks, how to control aggregate size, and observations on association strength. Using confocal microscopy, flow cytometry, and laser diffraction, they observed that, while very large (>1 mm) aggregates can form, they may also dissociate and reform. Aggregates were forced through a nylon mesh filter and observed afterward: as the filter size decreased, resultant aggregates were smaller. When the pore size was reduced to less than the diameter of an individual cell, the backpacks were still attached. This implied to them that the attachment is sufficiently strong such that the backpacks would remain attached to a lymphocyte undergoing extravasation *in vivo.* In conclusion, they feel that an injectable backpack system could have applications in lymphoid tissue engineering as described by others [52, 53], as well as more general cellular engineering applications requiring close cell association.

## 3. Challenges and Future Opportunities

In this section, challenges such as safety considerations and reformulation strategies to overcome loading limitations, overdosing, and clearance issues are addressed. The opportunities lie in the enhanced capabilities with respect to improves therapeutic intervention strategies and additional applications for nanomedicine in the healthcare sector. 

The perception that nanomaterials have inherent incompatibility issues with respect to the uptake into the human systemic environment has been addressed by many nanobiotechnology researchers (see Zook et al. [54] for a representative paper from the Biochemical Science Division of the National Institute of Standards and Technology).

Concerns such as toxicity, leaching, clearance, reproducibility/nonuniformity, chaperone characteristics/use of surface active agents and stability are major factors affecting the revolutionization of nanomedicine. The presence of multiple nanotechnology based drugs in the market place attests to the resolution of many of these issues. However, many more related to bioefficacy, loading capacity, and other features associated with performance optimization present ongoing challenges and opportunities for advances in nanomedicine thereby ensuring that it represents the future of medical care. General discussions, with key literature references, can be found in sources such as the Biomedical Engineering Handbook [55]. Of particular interest would be the section devoted to bionanotechnology with specific articles related to nanomaterials: perspectives and possibilities in nanomedicine [56]. The following comments are excerpts from their work and that of many other previously mentioned researchers [1–10, 31, 32, 35, 45, 52], along with summary statements from previous sections of this paper. 

Specific illness treatments via nanomedicine protocols each have unique detriments that can be remedied by providing a range of delivery systems. The concept is to develop methods of controlled therapeutic delivery and release to specific tissues and tumors over a desired timeline. These systems are designed specifically to deliver soluble drugs, proteins, vaccine adjuvants, and plasmid DNA for gene therapy by exposing target cells to their cargo. The chaperone is thus required to enter the cells via endocytic or phagocytic pathways and release its payload through degradation and diffusion mechanisms. The major challenge here is to accomplish these tasks while addressing the issues of biocompatibility, biodegradation, and the capture and clearance by the reticuloendothelial system (RES). Although excelling at some aspects, the current systems often fail to incorporate all required characteristics for high *in vivo* performance. 

The chaperones for therapeutic nanoentities include viral carriers, organic and inorganic nanoparticles, and peptides. Although the efficient targeted delivery of therapeutic drugs continues to present challenges (with tremendous potential benefits), the emerging research into proteomics, for gene therapy as the future of nanomedicine treatments is attracting more attention. Fortunately, the necessary gene transfection considerations are directly applicable to drug delivery systems also.

The current carriers used for transfection are mainly adeno- and retroviruses. Although highly efficient they pose immunogenic and mutagenic hazards which led researches to seek nonviral vectors. These include liposomes and nanoparticles of peptides and polymers, both synthetic and natural. Selection of vector type is dictated by the therapeutic agent, required pharmacokinetics, and the target cellular system, in addition to physical properties such as zeta potential (positive surface charge). The binding to blood proteins, clearance by the RES, and circulation times in the range of hours, rather than minutes, can be key performance targets/specifications. Hydrophilic polyethylene glycol (PEG) or longer chain polyethylene oxide (PEO) are commonly used synthetic polymers. Chitosan and alginate are useful natural polymers due to their excellent biodegradability characteristics. Biocompatible peptides show significant promise since they are able to bypass traditional endocytic pathways. Specific details can be found in Douglas et al. [56] and their accompanying literature references. The practical considerations enumerated there stress the need for the control of zeta potential, surface functionality via physical and chemical modifications, and the attainment of desired sizing. The method used to determine size is also important since dynamic light scattering (DLS) frequently gives larger measurement values than electron microscopy. Furthermore, DLS is particularly dependent on the presence of aggregate-inducing ions and proteins.

Vehicle surface characteristics are essential to control the contact time these vectors remain in the vasculature of a target region with respect to endocytosis and/or cargo release kinetics. Thus, in addition to chemical functionalization there exists numerous opportunities for magnetic, heat, and light affected systems influenced by external stimulus/fields.

These technological advances will translate into significant market enhancements. This is clear for both new and old drugs. For example, nanosizing of current marketed products is a means of providing these old drugs a new delivery platform offering new benefits and improved performance. FDA records indicate that the majority of approvals are reformulations or combinations of previously approved products. As a new candidate proceeds through its clinical testing program, it can be refined and/or postprocessed from its discovery formulation to meet the requirements of the emerging target product profile; that is, its delivery route, dosage, and pharmacokinetic behavior.

Considering its vast potential it becomes evident that nanotechnology will have a significant impact upon the drug delivery sector and its ability to provide sound technological solutions for drug development programs. Consequently, market expectations for the nanotechnology drug delivery platform are high, and it is estimated that it will increase to about $ 16 billion (USD) by 2014 [10].

## 4. Conclusions

Novel nanomaterial manufacturing methods and emerging nanotechnology applications for the pharmaceutical industry have been discussed in this paper. These manufacturing methods combine features such as bottom up nanoparticle formation for control of size and crystal structure with continuous manufacturing and Process Analytical Technology (PAT) for quality control and compatibility with the strict requirements imposed upon the pharmaceutical industry. The production of carefully engineered nanoparticles produced at high throughput rates and elevated technoeconomic stature demonstrates the role that transport phenomena has in path forward approaches for advanced drug delivery.

## Figures and Tables

**Table 1:** Current and emerging nanotechnology.

	Methods	Applications
Current	(i) Top down (ii) Batch manufacturing	(i) Enhanced bioavailability
		(ii) Delayed delivery
		(iii) Extended delivery
		(iv) Pulsitile delivery

Emerging		(i) Targeted delivery
	(i) Bottom up	(ii) Simultaneous targeted, imaging, and delivery
	(ii) Continuous manufacturing	(iii) Delivery to the brain (overcoming the Blood Brain Barrier)
	(iii) PAT	(iv) Delivery through novel targeting chaperons, (example T-cells)
		(v) Artificial organs, tissue therapy, wound healing, and so forth.

**Table 2:** List of various “bottom up” processes and influence on particle properties.

Bottom up processes	Properties controlled
Crystallization	(i) Size, shape
	(ii) Crystalline structure—Crystalline/amorphous
	(iii) Polymorph

Precipitation	(i) Size, shape
	(ii) Surface area

Encapsulation in polymers	(i) Size, shape
	(ii) API concentration
	(iii) Particle nanostructure

Chemical reactions	(i) Size, shape
	(ii) Purity
	(iii) Surface area
